# It’s TIME for a biomarker-driven approach to cancer immunotherapy

**DOI:** 10.1186/s40425-016-0147-8

**Published:** 2016-08-16

**Authors:** Leisha A. Emens

**Affiliations:** Department of Oncology, Johns Hopkins University School of Medicine, The Kimmel Cancer Center at Johns Hopkins, Bunting-Blaustein Cancer Research Building 1, 1650 Orleans Street, Room 409, Baltimore, MD 21231-1000 USA

Cancer immunotherapy has clearly taken its place in the clinic as one pillar of cancer care. Immune checkpoint blockade, which unleashes the activity of pre-existing T cells at the tumor site, is transforming cancer treatment. The FDA approval of the cytotoxic T lymphocyte antigen-4 (CTLA-4) antagonist ipilimumab for unresectable or metastatic melanoma on March 25, 2011 forged a path for the further clinical development of immune checkpoint modulation. Building on its success, antagonists of the programmed death-1 (PD-1) pathway have now been approved for use in advanced melanoma, non-small cell lung cancer (NSCLC), renal cell carcinoma, bladder cancer, and Hodgkin’s disease. Blockade of the PD-1 pathway produces durable objective responses in 20–30 % of patients with solid tumors, with clear activity in multiple other cancer types as well [1]. This striking clinical success has re-invigorated interest in cancer immunotherapy, promoted the rapid growth of the immuno-oncology pipeline, and accelerated the clinical development of treatment strategies that engage the immune system to treat cancer.

Therapeutic cancer vaccines, designed to promote the activation and expansion of T cells, have been studied in the clinic since the late 1800’s with mostly negative results. This lack of success has been attributed to both suboptimal vaccine formulations, flawed clinical development plans, and dominant mechanisms of immune tolerance and suppression in the tumor microenvironment that prevent T cell activity [2–4]. There was a flash of hope for therapeutic cancer vaccines on April 29, 2010 when the FDA approved sipuleucel-T for the treatment of asymptomatic or minimally symptomatic castrate-resistant prostate cancer. Sipuleucel-T is a cellular immunotherapy product composed of autologous peripheral blood mononuclear cells (PBMC) obtained by leukapheresis and activated with a recombinant fusion protein that delivers the tumor antigen prostatic acid phosphatase (PAP) linked to the immune-activating cytokine granulocyte-macrophage colony-stimulating factor (GM-CSF). The pivotal trial was a placebo-controlled multicenter trial that randomized 512 patients at a 2:1 ratio to receive three infusions of sipuleucel-T or unactivated control PBMC over the course of 1 month. It demonstrated a modest 4.1-month improvement in overall survival, where the sipuleucel-T-treated and control groups had a median overall survival of 25.8 and 21.7 months, respectively (HR = 0.775, 95 % CI 0.61–0.098, *p* = 0.032) [5]. There was no impact on progression free survival. Unfortunately, the complexity of the manufacturing process, the cost, and the rapidly changing landscape of prostate cancer therapy led to slow uptake of sipuleucel-T and the original manufacturer ultimately declared bankruptcy.

Despite these challenges, interest in cancer vaccines is growing. Quoix and colleagues recently reported promising results of the Phase 2b portion of the TIME trial, a Phase 2b/3 clinical trial testing the vaccine TG4010 combined with chemotherapy in patients with advanced NSCLC [6]. TG4010 is a recombinant modified vaccinia Ankara virus that delivers the tumor antigen mucin-1 (MUC-1) with the immune-activating cytokine interleukin-2 (IL-2). The induction of an immune response specific for MUC-1 is enhanced both by danger signals present in the viral vector and by local expression of IL-2 at the vaccine site. The Phase 2b/3 trial was based on promising results of earlier randomized trials demonstrating the safety and clinical activity of TG4010 in combination with chemotherapy in NSCLC [7, 8]. These trials identified a candidate predictive biomarker: TrPAL, activated natural killer cells triple positive for CD16, CD56, and CD69. A low baseline level of TrPAL appeared to predict for TG4010 activity in combination with chemotherapy; the 25 % of patients with the highest TrPAL values did not benefit, whereas the 75 % of patients with the lowest TrPAL levels did. High levels of TrPAL are thought to suppress the induction of an adaptive immune response by TG4010. This apparent conundrum may be explained by the dual role of natural killer cells in the regulation of adaptive immunity.

The TIME trial is a randomized, double-blind, placebo-controlled Phase 2b/3 trial that enrolls previously untreated patients with stage 4 NSCLC without a known activating *EGFR* mutation and with MUC-1 expression in at least 50 % of tumor cells [6]. In the Phase 2b portion, 222 patients were randomized 1:1 to receive 10^8^ plaque-forming units (pfu) of TG4010 or placebo (the formulation buffer of TG4010) from the initiation of chemotherapy weekly for 6 weeks, and then every 3 weeks until disease progression. Randomized patients were stratified according to the baseline level of TrPAL (≤ or > the upper limit of normal (ULN)). Chemotherapy was a platin-based doublet given at standard doses for up to 6 cycles, and bevacizumab and maintenance treatment with pemetrexed or erlotinib were permitted as clinically indicated. The primary endpoint was PFS assessed every 6 weeks to validate the predictive value of the TrPAL biomarker. For the population as a whole, the median PFS for the TG4010 and placebo groups was 5.9 months (95 % CI 5.4–6.7) and 5.1 months (95 % CI 4.2–5.9) respectively (HR = 0.74, 95 % CI 0.55–0.98, one-sided *p* = 0.019). The treatment effect of adding TG4010 to chemotherapy was delayed, consistent with reported data with other cancer immunotherapies like sipuleucel-T and ipilimumab [5, 9]. The study validated the association of the TrPAL biomarker with benefit in patients with a TrPAL value ≤ ULN, but not the association of the TrPAL biomarker with lack of benefit in patients with a TrPAL value > ULN. As this threshold corresponded to the first 3 quartiles (Q3) of the patient distribution (75 %) in the earlier Phase 2b trial [7], a pre-specified analysis demonstrated the patients with TrPAL values ≤ Q3 had a significant improvement in PFS, but those with TrPAL values > Q3 did not. Based on these data supporting an improvement in PFS with TG4010 with chemotherapy relative to control, and the predictive value of a low baseline level of TrPAL, the study continues to fully evaluate the clinical activity of TG4010 and to validate TrPAL as a companion predictive biomarker in this patient population.

Importantly, the TIME trial evaluated multiple biomarkers. First, it required expression of the tumor antigen MUC-1 in at least 50 % of tumor cells for eligibility. This is important as MUC-1 is the target of the vaccine-induced T cell response. Ensuring adequate levels of expression of the target antigen by the tumor is thus critical for success. Although not reported here, an association between the clinical activity of TG4010 and MUC-1-specific T cells in NSCLC patients has been reported in an earlier trial (8). Second, it evaluated the TrPAL biomarker as an additional predictive biomarker of benefit with the combination of TG4010 and chemotherapy. The investigators demonstrated that low levels of TrPAL appear to predict clinical benefit from TG4010 combined with chemotherapy. Third, in a post-hoc exploratory analysis, the investigators evaluated expression of PD-L1 in available pretreatment tumor specimens; 160 (72 %) patients were assessable for tumor cell PD-L1 expression, and 137 (62 %) patients were assessable for PD-L1 expression in infiltrating immune cells. They noted a significant improvement in PFS with TG4010 and chemotherapy relative to the control group in patients with a low level of PD-L1 expression in the immune cell infiltrate, but not in those with higher levels of PD-L1 expression in the immune cell infiltrate (HR 0.61 [95 % CI 0.39–0.96]; *p* = 0.015). The level of PD-L1 expression by tumor cells appeared to make no difference. This finding raises the interesting possibility that adding a PD-1 or PD-L1 antagonist to block intratumoral immune suppression in patients with higher levels of PD-L1 expression at the tumor site could expand the number of patients who respond to TG4010 combined with chemotherapy. Prospective clinical trials will be required to test these hypotheses.

Where are we with simple predictive biomarkers in cancer immunotherapy? The PD-1 antagonist pembrolizumab was granted accelerated approval by the FDA on October 2, 2015 for the second line treatment of advanced PD-L1+ NSCLC together with the companion diagnostic PD-L1 IHC22C3 pharmDx test. This approval was based on objective response rates of 45 % in PD-L1+ patients relative to 19 % in all patients enrolled on the trial [10]. Nivolumab and atezolizumab also appear to have greater activity in tumors that express PD-L1 [11–15], and nivolumab is also FDA-approved for the second line therapy of NSCLC. Separately, a diagnostic test for PD-L1 expression, the PD-L1 IHC 28-8 Pharm Dx test, was approved to help physicians select patients more likely to benefit from PD-1 blockade with nivolumab. Thus, PD-L1 expression within the tumor microenvironment appears to enrich for responders to antagonists of the PD-1 pathway, but is an imperfect biomarker due in part to geographic variability in expression within a given tumor and across tumor metastases, and dynamic changes in PD-L1 expression over time. Microsatellite instability (MSI) has more recently been proposed as a predictive biomarker of response to pembrolizumab based on response rates of 40 and 0 % in mismatch repair deficient and proficient tumors, respectively [16]. This is thought to reflect a higher load of neoantigens generated by mutation in the mismatch repair deficient tumors, rendering them more immunogenic. These findings are being further explored and validated in larger trials. With these advances, we are beginning to develop informative predictive biomarkers of response to cancer immunotherapies. An association between the baseline level of TrPAL and the response of MUC-1+ NSCLC to TG4010 and chemotherapy, if validated in the Phase 3 portion of the TIME trial, could add another promising predictive biomarker to our toolbox. Companion predictive biomarkers of response and resistance to cancer immunotherapies are important to identify the patients most likely to respond, thus avoiding unnecessary toxicity and cost in patients unlikely to benefit. Thus, elucidating reliable biomarkers of response and resistance to established and emerging cancer immunotherapies should continue to be a high priority for clinical development.
